# Delayed-onset biliary peritonitis after endoscopic ultrasound-guided hepaticogastrostomy for malignant distal biliary obstruction

**DOI:** 10.1055/a-2364-4298

**Published:** 2024-07-29

**Authors:** Hiroki Sakamoto, Hirotoshi Ishiwatari, Takuya Doi, Junya Sato, Hiroyuki Ono

**Affiliations:** 1 38471 Division of Endoscopy, Shizuoka Cancer Center, Sunto-gun, Japan

Endoscopic ultrasound-guided biliary drainage (EUS-BD) is performed when biliary drainage using endoscopic retrograde cholangiopancreatography (ERCP) is difficult 1 2 3 4 . Biliary peritonitis is an adverse event that can be fatal; however, it typically develops immediately after the procedure 5 . Metallic stents reduce the risk of peritonitis. However, plastic stents are preferred for patients with benign diseases or who are surgical candidates. Herein, we present a case of peritonitis that developed 6 days after EUS-BD ( Video 1 ).

Delayed-onset biliary peritonitis occurred after endoscopic ultrasound-guided hepaticogastrostomy (EUS-HGS) using a plastic stent in a surgical candidate. A biliary stent inserted antegradely through the HGS route facilitated additional drainage to manage the biliary peritonitis.Video 1

An 82-year-old man was admitted to our hospital with obstructive jaundice. Computed tomography (CT) revealed distal biliary obstruction and bile duct dilation, indicating distal biliary cancer ( Fig. 1 ). ERCP was first attempted; however, it failed, and EUS-guided hepaticogastrostomy (HGS) was performed. A 7-Fr plastic stent (Through & Pass Type IT; Gadelius Medical) was inserted from the stomach into the bile duct, because the patient was scheduled to undergo surgical resection ( Fig. 2 ). The postoperative course was uneventful, and the patient started eating meals.

**Fig. 1:**
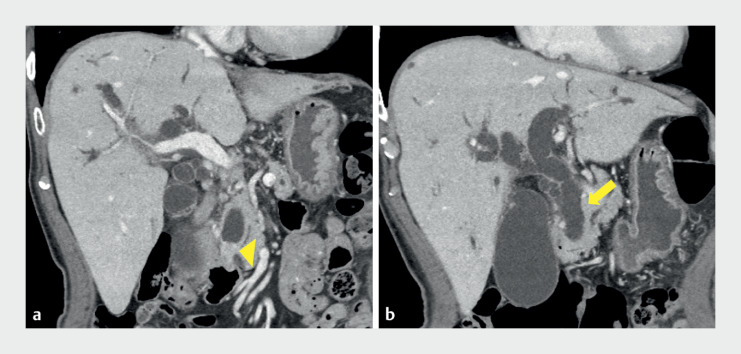
Computed tomography (CT) images on admission of an 82-year-old man with obstructive jaundice revealed: **a** distal biliary obstruction (arrowhead), and **b** dilation of the bile duct (arrow), indicating distal biliary cancer.

**Fig. 2:**
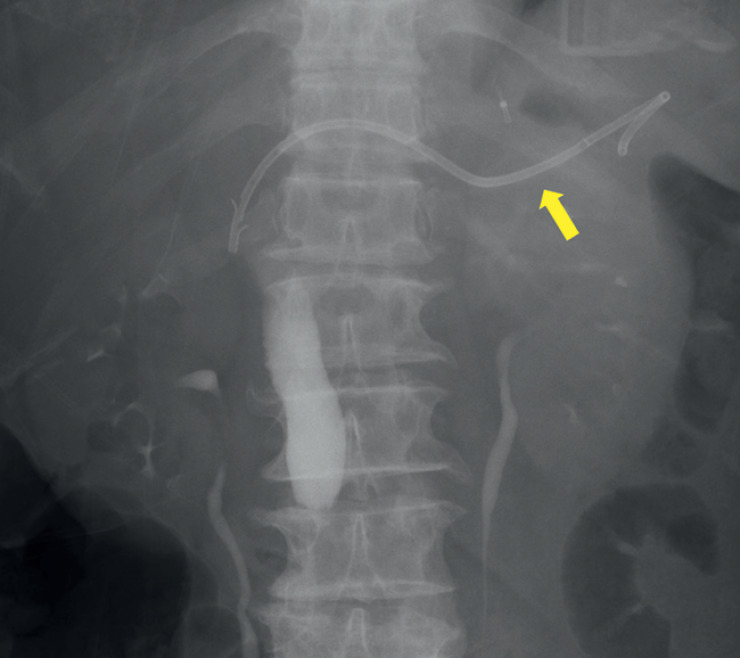
A 7-Fr × 14-cm plastic stent (Through & Pass Type IT; Gadelius Medical) was inserted from the stomach to the bile duct (arrow).

However, on the 6th day after EUS-HGS, he suddenly complained of upper abdominal pain and developed a high fever. CT revealed ascites around the liver, stomach, and spleen with the plastic stent remaining in place, raising suspicion of biliary peritonitis ( Fig. 3 ). After inserting a duodenal scope into the stomach, we saw food residue adhering around the stent, leading us to consider this to be the cause of stent obstruction that had occurred before maturation of the fistula ( Fig. 4 **a** ). An ERCP catheter and a guidewire were inserted into the bile duct alongside the stent, and the guidewire was passed beyond the distal biliary obstruction into the duodenum. Then a fully covered self-expandable metal stent (Braided 6; SB-Kawasumi, Japan) was placed antegradely across the distal biliary obstruction ( Fig. 4 **b** ). Subsequently, the patient’s peritonitis improved.

**Fig. 3:**
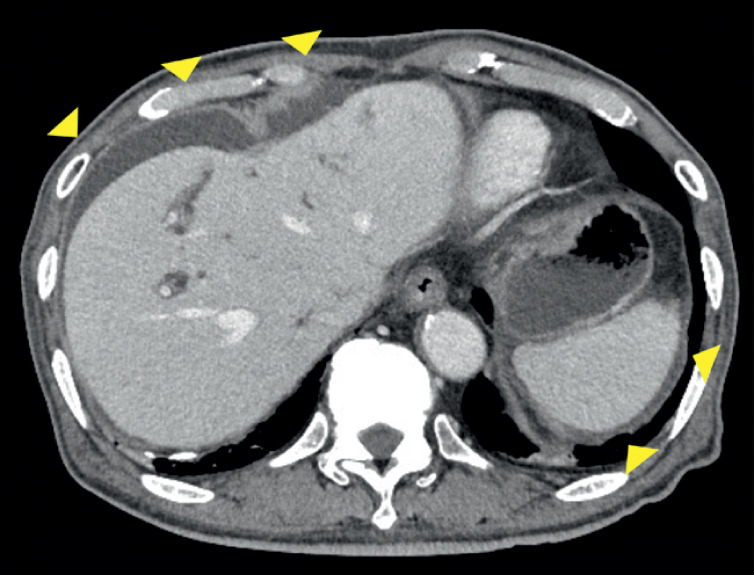
CT showed ascites around the liver, stomach, and spleen with the stent remaining in place, raising the suspicion of biliary peritonitis (arrowheads).

**Fig. 4:**
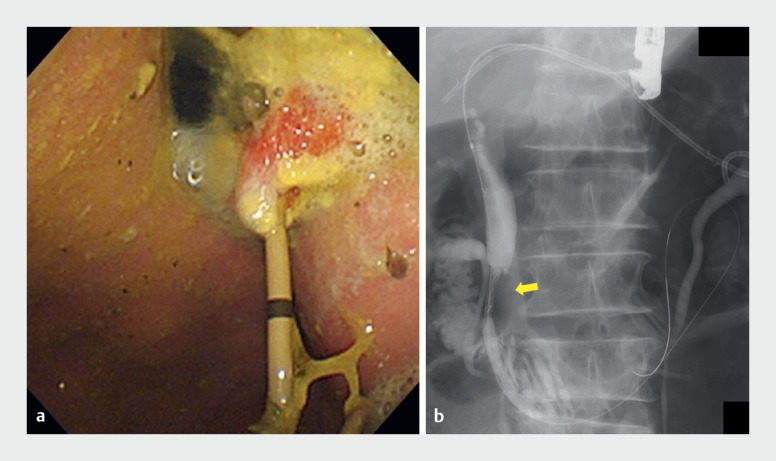
**a** After inserting a duodenal scope into the stomach, we observed food residue adhering around the stent, leading us to consider this to be the cause of stent obstruction that had occurred before fistula maturation. **b** A 6-mm × 10-cm fully covered self-expandable metal stent (Braided 6; SB-Kawasumi, Japan) was placed antegradely (arrow).

Endoscopists should be aware that peritonitis can occur before fistula maturation when a plastic stent is used. Antegrade stenting can be a treatment option for such cases.

Endoscopy_UCTN_Code_CPL_1AL_2AD

## Notes

Endoscopy E-Videos https://eref.thieme.de/e-videos
*E-Videos* is an open access online section of the journal *Endoscopy* , reporting on interesting cases and new techniques in gastroenterological endoscopy. All papers include a high-quality video and are published with a Creative Commons CC-BY license. Endoscopy E-Videos qualify for HINARI discounts and waivers and eligibility is automatically checked during the submission process. We grant 100% waivers to articles whose corresponding authors are based in Group A countries and 50% waivers to those who are based in Group B countries as classified by Research4Life (see: https://www.research4life.org/access/eligibility/ ). This section has its own submission website at https://mc.manuscriptcentral.com/e-videos .
